# Interoperable Integration of a National Rare Disease Registry Into a Rare Eye Disease Data Warehouse: Implementation Study

**DOI:** 10.2196/79378

**Published:** 2026-05-26

**Authors:** Camille Beluffi Marin, Marilyne Oswald, Laura Ratenet, Matthieu Stoll, Kirsley Chennen, Hélène Dollfus

**Affiliations:** 1 Laboratoire de Génétique Médicale, UMR_S INSERM U1112, 1 rue Eugène Boeckel, Strasbourg, 67000, France, 33 03 68 85 36 60; 2 FSMR SENSGENE, Coordination Center, Hôpitaux Universitaires de Strasbourg, Strasbourg, France; 3 FSMR SENSGENE, Centre de Référence Pour les Affections Rares en Génétique Ophtalmologique (CRMR CARGO), Institut de Génétique Médicale d'Alsace (IGMA), ERN-EYE, Hôpitaux Universitaires de Strasbourg, Strasbourg, France

**Keywords:** rare diseases, health data warehouse, data interoperability, secondary use of health data, BaMaRa (French national registry for rare diseases), extract-transform-load

## Abstract

**Background:**

In France, clinical data on rare diseases are primarily collected through BaMaRa (Base Maladies Rares), a software platform used by national expert centers to populate the BNDMR (Banque Nationale de Données Maladies Rares), the French national rare disease data warehouse. BaMaRa ensures standardized and structured data collection across all rare disease networks, with a focus on care coordination and epidemiological reporting. In 2024, FREDD (French Rare Eye Disease Database), a health data warehouse dedicated to rare eye diseases, was developed within the framework of the third French National Rare Disease Plan by the SENSGENE sector. Despite overlapping datasets, there is no native interoperability between BaMaRa and FREDD, requiring the development of a dedicated, traceable pipeline to transform BaMaRa exports into data suitable for inclusion in FREDD. This transformation involves complex business rules to address structural, semantic, and specific differences between the two systems.

**Objective:**

This study aims to describe the design and implementation of a robust data transformation pipeline that enables the automated conversion of BaMaRa clinical records into a structured dataset aligned with the FREDD data model. The primary goal is to ensure that the data remain semantically consistent and reusable for the secondary use of health data.

**Methods:**

We developed a Python-based application called FREDDEX that integrates several configuration files and encodes the domain-specific business rules required to align BaMaRa data with the FREDD schema. These rules include patient filtering, mapping of variable names and values, management of multisource redundancy, and prevention of overwriting. The system was designed to be modular, auditable, and usable by clinical data managers with minimal technical expertise.

**Results:**

FREDDEX was tested and validated on a BaMaRa export of 1000 real patients from Strasbourg University Hospital. The tool successfully filtered and created 641 patient profiles in FREDD, with a 99% success rate for attempted imports and full concordance (100%) for directly mapped and inferred variables. Genetic data reconstruction was confirmed on a random sample of 30 patients with genetic information, showing 100% accuracy, and multidiagnostic blocks were correctly handled in all manually reviewed cases. Beyond validation, FREDDEX processed up to 5000 patient records, enabling the rapid onboarding of new clinical sites and significantly reducing manual curation time, while runtime and memory usage demonstrated near-linear scaling. Importantly, the tool established a facilitated reproducible framework adaptable to other rare disease contexts and interoperable with national and European platforms, such as European Reference Network-EYE.

**Conclusions:**

This work demonstrates that transforming structured national rare disease registry data into a research-oriented health data warehouse is feasible when clinical business rules are explicitly formalized within an auditable extract-transform-load framework. Beyond the FREDD use case, this approach illustrates how interoperability between care-based and research infrastructures can be operationalized in rare diseases while preserving semantic integrity and regulatory compliance.

## Introduction

### The French Rare Diseases Ecosystem

Research on rare diseases relies heavily on available clinical data. However, by nature, rare diseases affect small, dispersed populations, making large-scale data collection inherently difficult [1]. Fragmentation across care centers, inconsistent data entry practices, and the absence of harmonized codification schemes further limit the availability of usable, research-grade data [2]. In France, however, rare diseases have been recognized early on as a major public health priority. Since 2005, 5 successive National Plans for Rare Diseases (PNMR) have enabled the structuring of a comprehensive network of expert clinical centers across the country. As part of the second PNMR (2011‐2016) [3], the BNDMR (Banque Nationale de Données Maladies Rares) was the first data warehouse dedicated to rare diseases, launched to centralize data collected by rare diseases expert centers, supported by the creation of BaMaRa (Base Maladies Rares), a software platform that collects standardized clinical information from these centers and transfers the data to the BNDMR. While BaMaRa plays a crucial role in national epidemiological monitoring and care coordination, it was not originally designed for downstream data reuse in research-oriented data warehouses [4-6].

The second PNMR led to the creation of rare disease specialized networks (23 networks of expert clinical centers specialized in specific groups of rare diseases), structuring furthermore the rare disease ecosystem [7]. In 2022, SENSGENE [8]—the French national rare disease sector for sensory disorders—identified the need to efficiently collect the data of patients with *rare eye disease* (RED). Taking the opportunity of the France 2030 call “Accelerating research and innovation on rare diseases through scientific databases” during the third PNMR and the RaReTiA project, SENSGENE clinicians gathered to create FREDD (French Rare Eye Disease Database) [9]. This health data warehouse (HDW) was designed as a research database to support the secondary use of clinical data from patients with RED. FREDD provides a structured and semantically enriched environment tailored to the needs of translational research, cohort building, and data reuse across multiple centers. In addition, the data collected in FREDD will be valuable for projects leveraging artificial intelligence, particularly in the field of medical imaging. Indeed, recent advances have demonstrated the potential of artificial intelligence to significantly improve the diagnosis, prognosis, and treatment of RED, especially through the analysis of multimodal data, including retinal imaging and optical coherence tomography [10-12].

### The Interoperability Gap Between BaMaRa and Research Data Warehouses

As a rare disease, HDW, a significant portion of the information hosted in FREDD naturally originates from expert clinical centers and overlaps with the data already collected in BaMaRa, including the Minimum Rare Disease Dataset (Set de Données Minimum Maladies Rares; SDM-MR). Rather than asking clinicians to manually reenter these data into FREDD, which would be time-consuming for overwhelmed health care professionals and burdensome for expert centers, the optimal approach is to directly retrieve the data collected in BaMaRa. This not only minimizes the risk of inconsistencies, fragmented datasets, and misaligned updates across systems but also streamlines the process and ensures that the data are consistently up to date.

This local redundancy reflects a broader structural issue in the rare disease data ecosystem: the lack of seamless interoperability between clinical data collection tools and research-oriented data warehouses. In response to this broader challenge, several national and European initiatives aim to improve interoperability between rare disease registries. First, REDgistry [13], a registry within the European Reference Network (ERN) for RED (ERN-EYE) [14,15], consolidates and harmonizes RED data across Europe and is planned to be connected to FREDD. The JARDIN project [16] aims at standardizing rare disease data at the national level, aligning with international interoperability standards. Complementing these efforts, the French Health Data Hub [17] provides secure support for the reuse of health data in research and public interest projects and serves as a national infrastructure to promote secure, General Data Protection Regulation (GDPR)–compliant data sharing.

**Figure 1.**
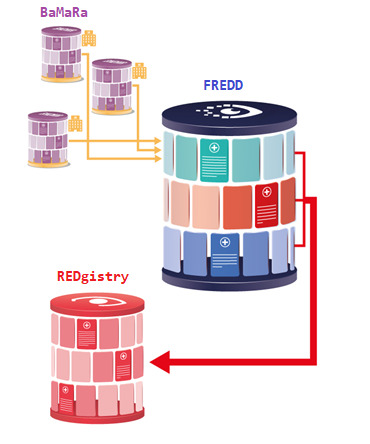
National and international landscape of rare eye disease (RED) data. BaMaRa: Base Maladies Rares; FREDD: French Rare Eye Disease Database.

Together, these initiatives establish a strategic and regulatory framework for rare disease data interoperability within the national and European ecosystem (Figure 1). However, they do not directly resolve the technical challenge of transforming and transferring structured data between heterogeneous information systems in routine clinical practice. To address this operational gap, we developed FREDDEX, a data integration application that automates the extraction, transformation, and loading of BaMaRa data into FREDD, implementing explicit business rules derived from the BaMaRa’s clinical logic.

This paper presents the architectural design and methodological framework of FREDDEX, detailing its rule-based transformation logic and technical implementation. Using FREDD as a real-world case study, we demonstrate the feasibility of structured BaMaRa data reuse within a research-oriented HDW and discuss the challenges encountered when translating complex clinical logic into computable rules. To our knowledge, this represents the first application developed to establish an operational link between BaMaRa and a research database other than the BNDMR.

## Methods

### BaMaRa: Technical Overview

#### Dataset and Electronic Record

BaMaRa is a web-based application developed by Assistance Publique–Hôpitaux de Paris [18] and provided free of charge to partner health care institutions. It supports both manual data entry by clinicians and automated data extraction from hospital electronic health records via interoperability standards, with both approaches often used complementarily to complete missing information.

The underlying data model is based on the SDM-MR, initially comprising around 60 mandatory items but has currently expanded to over 120 variables due to technical and structural extensions. These variables are organized into domain-specific forms within the user interface, in accordance with the BNDMR dataset structure, and each patient is assigned a local identifier (BaMaRa ID) for site-level management.

Due to the coexistence of multiple data entry modes and the involvement of different clinicians, BaMaRa may generate multiple diagnostic blocks for a single patient rather than updating existing entries. These parallel entries reflect the accumulation of clinical information over time and may result in redundant or partially overlapping diagnostic data in exported datasets.

#### Data Export and Interoperability

Currently, BaMaRa offers data export functionalities exclusively in Excel format, following the structure defined by the BNDMR dataset and distributed across multiple worksheets. Each Excel sheet corresponds to the BaMaRa clinical domain tabs, with correspondence between sheets ensured through the BaMaRa ID. Although the underlying data model is technically structured according to the Health Level Seven International Clinical Document Architecture standard and relies on international reference terminologies, such as Orphanet [19], human phenotype ontology (HPO) [20], and the HUGO Gene Nomenclature Committee [21], this semantic structure is not directly preserved in a machine-readable form in the Excel export.

Indeed, the BaMaRa export is designed for human readability rather than system interoperability. Column headers consist of natural-language labels rather than standardized metadata identifiers. For example, a column may be explicitly named “*Origine de l’ADN*” (DNA origin) and contain free-text values such as “*nucléaire*” (nuclear) or “mitochondrial,” including spaces, accents, and heterogeneous spelling conventions. These labels do not follow controlled vocabularies, machine-readable schemas, or technical naming conventions.

Furthermore, the export does not embed formal metadata definitions, explicit data typing, or harmonized value coding. As a result, the dataset cannot be directly reimported into another information system without prior restructuring. Substantial transformation steps—including column normalization, metadata standardization, value recoding, and semantic alignment—are therefore required to enable integration with external databases such as FREDD.

#### FREDD: Dataset, Mapping, and Interface

##### Dataset Overview

**Figure 2.**
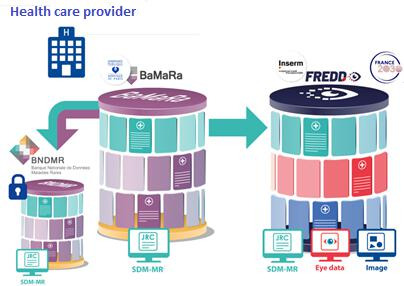
Conceptual relationship between the Banque Nationale de Données Maladies Rares (BNDMR), BaMaRa (Base Maladies Rares), and the French Rare Eye Disease Database (FREDD) dataset model, highlighting the shared Minimum Rare Disease Dataset (Set de Données Minimum Maladies Rares; SDM-MR) core variables and ophthalmology-specific extensions.

The dataset model used in FREDD extends the SDM-MR by incorporating ophthalmology-specific variables while preserving a substantial structural overlap between the 2 systems, as illustrated in Figure 2.

It contains 448 variables across multiple categories, of which 281 are potentially retrievable from BaMaRa (Table 1). Accounting for duplicated blocks across related sections (eg, main vs additional diagnosis), 83 unique BaMaRa variables can ultimately be extracted and mapped to FREDD.

**Table 1.** Overview of FREDD^a^ variables and their importability from BaMaRa^b^.

FREDD dataset category	Variables (total)	Variables retrieved from BaMaRa
Legal	7	0
Administrative	18	10
Status	4	4
Clinical diagnosis (1 main diagnosis and 1 potential additional diagnosis)	61	59 (diagnosis-related: 29; other: 1)
Genetic diagnosis (3 potential genes with each 3 potential variants, 1 main diagnosis, and 1 potential additional diagnosis)	196	170 (diagnosis-related: 85; gene-related: 3; variant-related: 8; other: 4)
Disease history (1 main diagnosis and 1 potential additional diagnosis)	28	22 (diagnosis-related: 10; other: 2)
Visual acuity exam	29	0
Visual field exam	22	0
Treatments	6	3
Disability	7	0
Relevant medical history	31	5
Clinical protocol (3 potential protocols)	13	10 (protocol-related: 3; other: 1)
Image deposit	25	0

a FREDD: French Rare Eye Disease Database.

b BaMaRa: Base Maladies Rares.

##### Variable Mapping

The 83 unique BaMaRa variables were classified into 3 types based on the method of derivation: directly mapped, inferred, or reconstructed from genetic data (Table 2).

**Table 2.** Examples of variables retrieved from BaMaRa^a^ and mapped to FREDD^b^.

Nature of retrieved variables	Count
Directly mapped	54 (18 use reference terminologies)
Inferred	17
Reconstructed from genetic data	12

a BaMaRa: Base Maladies Rares.

b FREDD: French Rare Eye Disease Database.

Directly mapped variables rely on the predefined BaMaRa → FREDD mapping file. FREDDEX reads the BaMaRa export and automatically applies these correspondences to populate FREDD. For example, categorical values from BaMaRa can be automatically translated into the corresponding FREDD encoding scheme, such as converting “Affected” to 1 and “Not affected” to 0 in the “adm_patient_MR” field. If a variable has no defined correspondence in the mapping file, it is imported as-is into FREDD without transformation and is subsequently reviewed during the FREDD data management process to ensure consistency and semantic alignment.

The 17 inferred variables are derived through additional calculations applied to BaMaRa values, with default values assigned when source data are missing or inconsistent. The 12 reconstructed variables relate to genetic data, which are flattened in FREDD to maintain patient-diagnosis-gene-variant relationships in a single-level structure.

Controlled terminologies are enforced by parsing the XML files of each reference system, ensuring that all values match FREDD’s validation rules. Unmapped or invalid values, such as nonexistent HPO or ORPHA codes, trigger errors and prevent patient data from being imported, preserving semantic integrity.

##### Implementation of Domain-Specific Business Rules

FREDD is designed as a cross-sectional research registry and does not support longitudinal tracking or iterative updates of previously imported patient records. Therefore, each patient is intended to be imported once, with a dataset reflecting the most up-to-date validated clinical state at the time of transfer. Consequently, FREDDEX must determine whether it is relevant to import a patient, and which data elements should be retained.

In addition to deterministic mapping, FREDDEX incorporates a set of domain-specific business rules to manage BaMaRa data consistently and ensure semantic coherence when populating FREDD. These rules, summarized in Table 3, address patient selection, duplicate prevention, multiple diagnostic blocks, and polydiagnostic management. They are designed to handle known sources of complexity in the BaMaRa data entry.

**Table 3.** Overview of domain-specific business rules in FREDDEX.

Rule category	Objective	Implementation logic
Patient selection and filtering	Restrict import to clinically allowed RED^a^ patients	ORPHA code must belong to a predefined configuration list; diagnostic status must be “Probable,” “Confirmed,” or “Undetermined” with ≥1 HPO^b^ term
Duplicate import prevention	Prevent overwriting curated data	Identity check using name, surname, and date of birth; existing patients are not reimported
Multiple diagnostic blocks	Resolve duplicated or overlapping BaMaRa^c^ diagnostic entries	Identification of multiple blocks; retention of the most recent block with updated values replacing previous ones while preserving earlier values when no newer information is available
Polydiagnostic management	Preserve distinct rare diseases for the same patient	Separate FREDD^d^ entries created for each eligible RED

a RED: rare eye disease.

b HPO: human phenotype ontology.

c BaMaRa: Base Maladies Rares.

d FREDD: French Rare Eye Disease Database.

Key rules implemented in FREDDEX include the following:

1. Duplicate import prevention: Before any creation request, FREDDEX queries the FREDD registry via the SKEZIA application programming interface (API) to determine whether a patient profile already exists. At this stage, the API provides access only to patient profile metadata and not to associated questionnaires or clinical content. As the profile schema contains only surname, given name, and date of birth as identifying attributes, identity matching is performed using these fields. If a matching profile is detected, the import is aborted for this patient to prevent duplicate entries or unintended overwriting of curated data. The matching algorithm is robust to compound first names (eg, “Marie-Anne” vs “Marie Anne”), which are correctly recognized as identical.
2. Patient selection and filtering: Only patients with an ORPHA code corresponding to RED (predefined in collaboration with the SENSGENE network) are retained. Patients are included if their diagnostic status is “probable” or “confirmed,” in which case an ORPHA code is required, or “undetermined” with at least 1 HPO code, for which an ORPHA code is not mandatory. This strategy ensures that only patients at a stage of their clinical journey where meaningful data can be captured are imported into FREDD.
3. Multiple diagnostic blocks: Patients may have multiple diagnostic blocks recorded in BaMaRa for the same RED, reflecting successive entries or updates within a single center. As FREDD cannot accommodate multiple successive states of the same diagnosis for a single patient, FREDDEX consequently identifies all diagnostic blocks corresponding to the same disease and retains only the most recent. When newer blocks provide updated values, these replace the previous ones; however, if no updated value is available for a given field, the previously recorded value is preserved to avoid information loss.
4. Polydiagnostic cases: When 2 distinct REDs are reported for the same patient, both are represented in FREDD as separate diagnostic entries.

Each rule is implemented as a discrete function within the transformation module and fully documented for audit purposes. The ORPHA code filter is managed through an external configuration file containing the allowed ORPHA codes, enabling center-specific customization of patient selection.

The log report provides structured feedback on the application of the business rules, including the identification of patients not imported, the handling of multiple diagnostic blocks (with specification of the retained variables), and the detection of polydiagnostic cases.

##### FREDD Electronic Record and API

The FREDD data collection relies on SKEZIA, a certified electronic case report form (e-CRF) solution developed by SKEZI [22], compliant with French health data regulations, for structured data collection. SKEZIA is used in clinical observational studies, such as the SPOON project [23], which focuses on generalized myasthenia gravis in France, showcasing its ability to collect standardized data at a scale. It supports modular form design, version control, and validation rules and allows precise configuration of each data entry field (types, units, and permissible values). The design, maintenance, and update of the FREDD e-CRF—such as the definition of data elements, the implementation of consistency rules, and the management of form versions—are overseen by the FREDD team, notably its data manager. The SKEZI team is involved in software upgrades and provides user support.

SKEZIA enables the use of controlled vocabularies (including ORPHA codes, HPO terms, Anatomical Therapeutic Chemical Classification medication [24] ontologies) and supports 2 secure data import mechanisms: a file-based import system that accepts CSV or Excel files formatted according to downloadable templates and a configurable API that allows for the automated transmission of structured data directly into patient questionnaires.

### Ethical Considerations

#### Ethics Approval

This study involved the development and performance evaluation of FREDDEX, a software tool for transforming and importing existing clinical data from BaMaRa into the FREDD registry. No new patient recruitment or prospective intervention was conducted, and no direct human subject experimentation was performed.

The study was based exclusively on secondary use of preexisting data extracted from the BaMaRa clinical database, operated under the regulatory framework of the BNDMR and subject to authorization by the French data protection authority (Délibération n°2019-113) [25]. Data processing within BaMaRa is conducted under institutional agreements and in compliance with applicable European and French data protection regulations, including the GDPR (Regulation [EU] 2016/679) [26] and the French Data Protection Act (Loi Informatique et Libertés) [27], with implemented organizational and technical safeguards, ensuring confidentiality and data security.

The FREDD database has also received Commission Nationale de l’Informatique et des Libertés (CNIL) authorization for health data processing (decision DT-2024-008 dated April 6, 2024) [28].

According to national regulations governing secondary use of health data and institutional data warehouse research, this type of retrospective study using pseudonymized data falls under the scope of approved data processing activities and does not require additional patient informed consent. Nevertheless, data processing is performed under strict governance, ensuring respect for confidentiality, traceability, and data minimization principles.

Institutional review board or ethics committee approval was not required for this work due to its retrospective, noninterventional design using pseudonymized secondary data within an authorized national health data warehouse framework, in accordance with applicable French and European regulations.

#### Regulatory Compliance and Pseudonymization

From a regulatory perspective, no structural legal barrier exists to data transfer from BaMaRa to FREDD. Each participating clinical center acts as the data controller for its own BaMaRa database, which contains only the data of patients managed within that center. Consequently, the transfer of these data into the FREDD e-CRF—implemented at the level of the same institution or under its responsibility—does not constitute an unauthorized third-party disclosure but rather an organized reuse within the existing governance framework.

To ensure transparency, patient information documents used in BaMaRa were updated to explicitly state that collected data may be transmitted to the national BNDMR infrastructure and, where applicable, to FREDD for specific purposes. This process is fully compliant with applicable regulations, GDPR, and the CNIL guidelines [29], under the existing legal and ethical frameworks governing both BaMaRa and FREDD. Detailed regulatory and governance aspects, however, fall outside the scope of this methodological publication.

In this context, no pseudonymization is applied prior to data transfer. BaMaRa data are directly identifiable within each clinical center and are transferred to FREDD in their original form under the responsibility of the same data controller. Pseudonymization is subsequently implemented within FREDD at the level of the e-CRF, where identifying elements are separated from clinical data and replaced with study-specific identifiers.

#### Data Partitioning and Center-Specific Access Control

These regulatory and governance principles directly determine the architectural design of FREDD: each clinical center contributing to FREDD operates within its own isolated data space. Through the SKEZIA e-CRF interface, a center can view, edit, and manage only the data it has entered; no access is granted to records entered by other centers. This segregation is ensured during automated data imports via the SKEZIA API through the generation of center-specific authentication tokens. Each token contains parameters uniquely identifying the target institutional workspace, thereby restricting API-based data ingestion to the corresponding dataset.

### Design Requirements and Constraints

While the Excel-based exports represent a concrete source of operational constraints, additional considerations influenced the overall design principles of FREDDEX. These constraints guided the technical implementation and shaped the system architecture to align with clinical deployment environments. To meet these needs, FREDDEX was designed around several key principles:

- User-friendliness: The tool can be operated by clinical trial technicians with limited programming experience and interacted through a very simple graphical user interface.
- Local execution and data security: FREDDEX runs entirely on the user’s workstation, without requiring external servers or remote hosting. BaMaRa exports are processed locally, and no patient-level data are transmitted outside the hospital information system. The tool is compatible with standard hospital workstations and operates within existing security policies and user permission frameworks.
- Extensibility: The tool is modular, allowing adaptation to future changes in the BaMaRa dataset or the FREDD data model.
- Error handling and support: FREDDEX generates detailed log files and error reports to facilitate technical support and enable the rapid resolution of integration issues.
- Transparency and auditability: The Python processing engine is extensively documented and generates detailed execution logs, providing dataset-level traceability of all applied transformation rules and facilitating reproducibility and auditability.

These constraints guided the technical design of FREDDEX, ensuring that the tool is functional, deployable, and maintainable in a clinical setting. The application was fully designed and implemented by the FREDD team, enabling controlled evolution and deployment across participating clinical centers.

**Figure 3.**
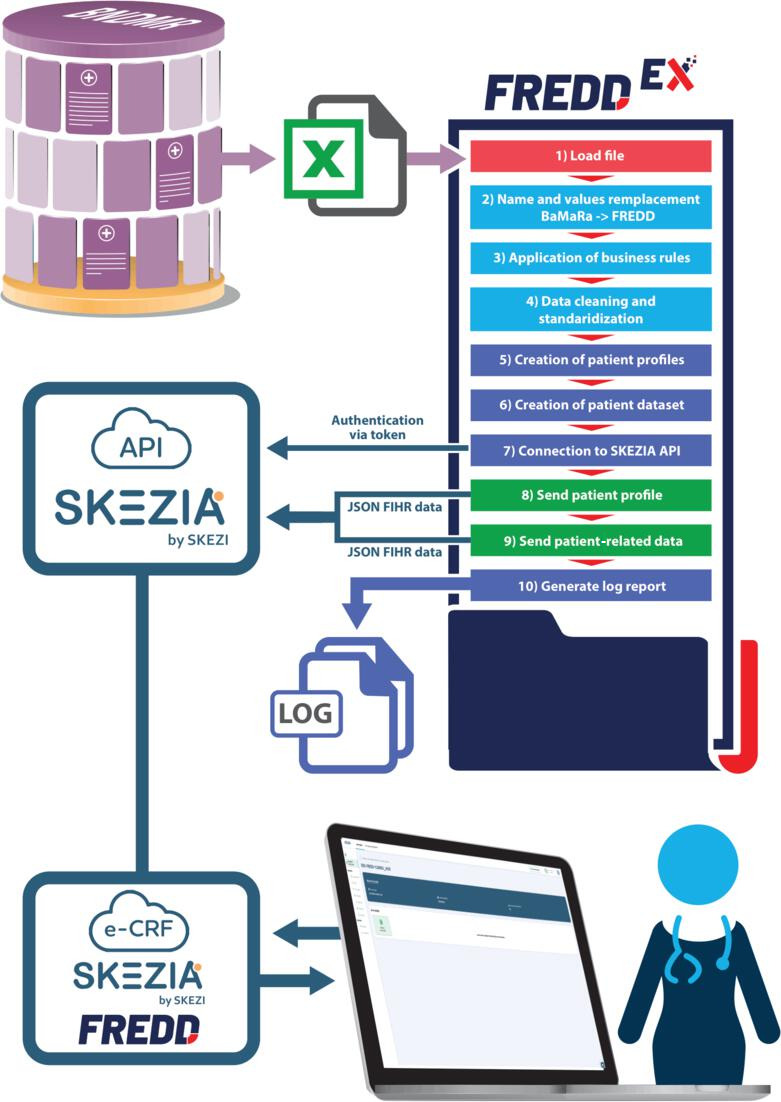
Diagram illustrating the main functions of FREDDEX. These functions correspond to the “extract” (in red), “transform” (in light blue), and “load” (in green) steps, or (in dark blue) to the application’s technical operations. API: application programming interface; BaMaRa: Base Maladies Rares; e-CRF: electronic case report form; FHIR: Fast Health Care Interoperability Resources; FREDD: French Rare Eye Disease Database.

FREDDEX follows a classical extract-transform-load architecture, implemented in Python and structured into 3 functional layers as detailed in Figure 3:

1. Extraction layer (in red): FREDDEX takes as input standardized Excel exports generated manually from BaMaRa by the local data manager. These files constitute the raw input layer and preserve the structural organization of BaMaRa clinical domains across multiple worksheets.
2. Transformation layer (in light blue): The transformation process combines deterministic structural mapping and domain-specific logic. It comprises the following:
  - structured ingestion of Excel data
  - deterministic mapping based on a predefined BaMaRa→FREDD correspondence file
  - the application of domain-specific business rules (eg, multiple diagnostic blocks, polydiagnostic cases, and conditional logic for optional variables)
  - data cleaning, normalization, and semantic alignment
  - reformatting into a structure compliant with SKEZIA API specifications and a Fast Health Care Interoperability Resources (FHIR) [30] based on the JSON schema.
3. Loading layer (in green): Once validated, the transformed dataset is serialized into a standardized CSV structure for FREDD import. For API-based ingestion, data are translated into FHIR-compliant resources and transmitted to the SKEZIA platform via authenticated API calls. Patient creation and questionnaire submission are handled sequentially to preserve relational integrity. Authentication relies on short-lived access tokens generated through a secure end point, with credentials encrypted using the Fernet algorithm (AES-128-CBC with HMAC-SHA256 authentication) [31] and decrypted only at runtime. To ensure robustness, the pipeline incorporates controlled submission pacing and retry mechanisms in case of temporary API unavailability.

After each execution, FREDDEX generates a structured report detailing imported patients, applied business rules, selected diagnostic blocks, and rejected or excluded records, thereby ensuring full transparency and auditability.

Having fully designed and implemented FREDDEX’s architecture and processing workflow, we next assessed its performance and reliability by validating the software on real-world BaMaRa data.

### Validation Dataset, Experimental Design, and Performances

To validate the entire data integration pipeline, a BaMaRa export file containing 1000 real-world patients from the Centre des Affections Rares Génétiques et Ophtalmologiques (CARGO) in Strasbourg was used for validation. The dataset was derived from routine clinical consultations and represented real-world clinical documentation.

The file was processed using FREDDEX and imported into a dedicated test instance of the FREDD e-CRF. After import, all patient records were reexported for comparison with the original BaMaRa dataset to assess mapping consistency and transformation behavior.

A structured validation protocol was applied to evaluate deterministic transformations. Automated checks were performed on directly mapped and inferred variables. Complex relationships between diagnoses, genes, and variants were evaluated on a random sample of 30 patients with genetic data, independently reviewed by a research assistant. Additional validation was performed on 30 patients with multiple diagnoses to assess correct handling of longitudinal clinical information. Duplicate import prevention was assessed by reprocessing a subset of 10 patients after the initial successful import.

To evaluate the software performance and scalability, runtime was measured from the opening of the FREDDEX interface to the display of the “Processing completed” window, while peak memory usage was monitored using the Python *psutil* library. After each major processing function, the resident memory size of the running process was recorded, and the highest observed value during execution was retained as the peak memory usage.

## Results

### Software Validation

From the BaMaRa export of 1000 real patients from the CARGO cohort in Strasbourg, FREDDEX successfully filtered eligible patients and created 641 patient profiles in the FREDD e-CRF, corresponding to the number of questionnaires imported without errors. Seven questionnaires failed to import due to mapping errors in an HPO field. Among the 641 imported patients, 9 had ORPHA codes not listed in the configuration file. These patients were still imported because they met the alternative inclusion criterion: a diagnostic status of “Undetermined” with at least one HPO code recorded. The transformation from BaMaRa to FREDD was validated by comparing the exported files from both systems. All directly mapped and inferred variables showed 100% concordance.

Among the 641 imported patients, 129 had at least one gene with one associated variant recorded. A random sample of 30 patients was manually reviewed to ensure that the correct genes were assigned to the appropriate diagnoses and that variants were correctly linked to their respective genes. This review confirmed 100% concordance.

Out of the 641 imported patients, 95 had at least 2 diagnostic blocks. Among these, 25 patients had multiple REDs, while the remaining 70 patients had multiple blocks corresponding to a single disease.

- Patients with multiple diagnostic blocks: For the 70 patients with multiple blocks for a single disease, FREDDEX retained the most recent and most complete block. A manual review of 30 patients confirmed 100% concordance.
- Patients with multiple diagnoses in FREDD: Of the 25 patients with multiple rare diseases, only 7 had 2 RED diagnoses recorded. The remaining diagnoses were excluded due to either an unauthorized ORPHA code or a diagnostic status not meeting inclusion criteria. For each retained block, all variables were verified to ensure accurate transfer and representation in FREDD.
- Duplicate import prevention: On all 10 patients deliberately reprocessed after the successful initial import, the second import attempt was correctly blocked. Existing records were identified based on identity criteria (name, surname, and date of birth), and no duplication or unintended overwriting occurred.

These results are summarized in Table 4.

**Table 4.** Success rates and error tracking for BaMaRa^a^-to-FREDD^b^ data transformation.

Patients concerned	Step	Success	Errors or exclusions	Success rate of eligible patients (%)	Cause
1000	Filtering per ORPHA/diagnostic status	648	0	100	352 patients excluded: only those with valid ORPHA+ probable/confirmed or undetermined+ HPO^c^ included
648	Initial import into FREDD	641	7	98.9	7 patients failed due to HPO mapping errors
641	Directly mapped and inferred variables	641	0	100	Concordance checked by code between BaMaRa and FREDD
30 randomly selected patients (out of 129 with ≥1 gene and variant)	Genetic data reconstruction	30 (manual review)	0	100	Random sample of 30 manually checked; correct genes/variants assigned to correct diagnosis
30 randomly selected patients (out of 95 with ≥2 diagnostic blocks)	Multidiagnostic blocks	30 (manual review)	0	100	Manual review of 30 patients confirms the most recent block correctly represented
25	Patients with multiple rare diseases	7 (manual review)	0	100	Remaining 18 excluded due to ORPHA/status rules; all manually verified
10	Duplicate import prevention (reimport test)	10	0	100	Second import attempt correctly rejected; identity matched on name, surname, and date of birth

a BaMaRa: Base Maladies Rares.

b FREDD: French Rare Eye Disease Database.

c HPO: human phenotype ontology.

### Performances Validation

**Figure 4.**
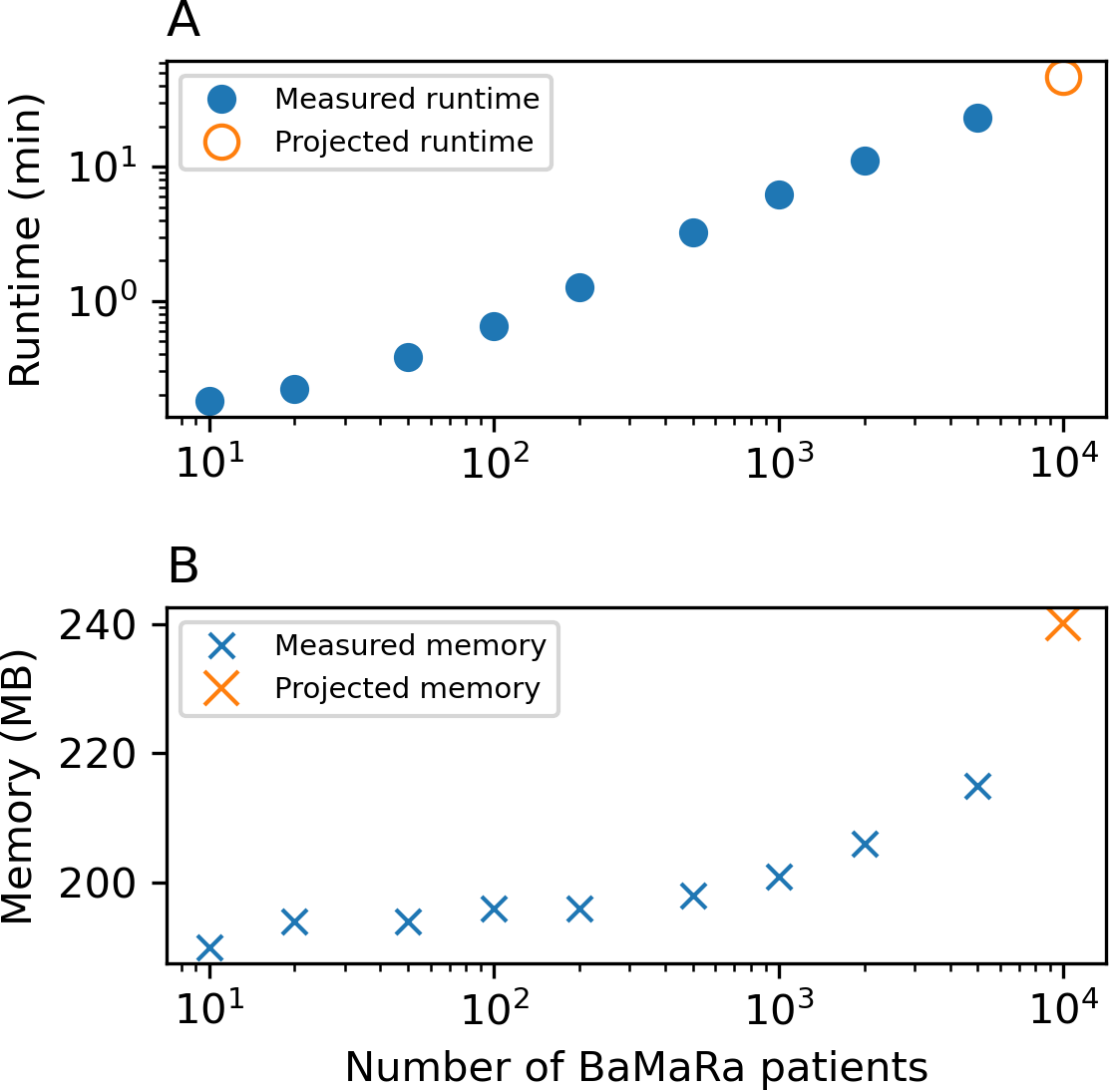
.Performance evaluation of the pipeline. (A) Runtime as a function of the number of BaMaRa (Base Maladies Rares) patients (log-log scale). (B) Memory consumption during execution. Orange markers indicate projections for 10,000 patients.

Performance measurements were conducted on datasets ranging from 10 to 5000 patients. The results indicate that execution time grows near-linearly with the number of patients (Figure 4A), while peak memory consumption remains stable between 190 and 215 MB (Figure 4B). Based on extrapolation from observed measurements, processing 10,000 patients is estimated to require approximately 46 to 47 minutes of runtime, with memory usage remaining within the observed range.

## Discussion

### Performance Evaluation of FREDDEX in Large-Scale Clinical Data Integration

In this study, FREDDEX successfully transformed and integrated the BaMaRa data into the FREDD data warehouse, generating 641 patient profiles from an initial dataset of 1000 patients and achieving a 99% success rate with full concordance for mapped and inferred variables. The tool also accurately reconstructed genetic data and handled complex clinical cases, including multiple diagnostic blocks, while demonstrating near-linear scalability. The development of FREDDEX represents a significant step toward harmonizing clinical data flows between routine care and research infrastructures, supporting the reuse of health data while reducing the burden on clinical centers.

### Bridging Clinical and Research Infrastructures

One of the main achievements of this work is its ability to operationalize the concept of interoperability in the rare disease ecosystem. While BaMaRa was not initially designed with research reuse in mind, its widespread adoption across expert centers makes it a key asset in the national rare disease data landscape. FREDD, as a domain-specific data warehouse, provides the necessary structure and enrichment layers to enable high-quality research on a specific field of RED. The integration tool serves as a bridge between these 2 complementary infrastructures, fostering data continuity without duplicative entry.

This approach aligns with broader national and European strategies for rare disease data management, including the REDgistry platform and the upcoming European Health Data Space, both of which emphasize data standardization and reuse. Beyond the technical alignment, the deployment of this application demonstrates that secure, regulation-compliant data transfers between clinical databases are feasible and can significantly preserve valuable human resources in expert centers. Moreover, the implementation of BaMaRa-specific business rules allows the tool to handle the complex realities of clinical data capture with nuance and flexibility. These rule-based transformations are key to ensuring semantic coherence and avoiding misinterpretation during downstream data use.

However, such integrations would be vastly simplified if a dedicated data entry software for rare diseases existed at the European level, built natively on the SDM-MR and embedding essential reference ontologies, such as ORPHA codes, HPO, and HUGO Gene Nomenclature Committee. In such a scenario, the “transform” step of extract-transform-load processes would become minimal or even obsolete, substantially easing the burden on technical teams managing rare disease data infrastructures.

More broadly, this highlights the need for public policy to support not only semantic and legal harmonization efforts but also concrete operational infrastructures—such as shared data entry tools—capable of ensuring sustainability, technical interoperability, and equity of access across national health systems. Strengthening these foundations is essential to fully realize the promise of federated rare disease data platforms in Europe.

### Flexibility, Interoperability, and Versioning

FREDDEX is built on a modular architecture with clearly compartmentalized components responsible for data ingestion, semantic mapping, transformation, and API export. This modular design ensures the independent operation of each stage, facilitating maintenance, debugging, and future enhancements. The BaMaRa → FREDD mapping is maintained externally from the source code, allowing variables to be added, removed, or modified without altering the core application logic. Similarly, the API layer is fully decoupled from upstream processing, enabling flexible integration with different export targets and supporting future alignment with common data models, such as FHIR or Observational Medical Outcomes Partnership.

To ensure reproducibility and traceability, FREDDEX follows a strict versioning strategy. A new version is released whenever bug fixes, user-interface improvements, or adjustments due to updates in BaMaRa or the SKEZIA API are required. Each version is distributed with instructions to discontinue the previous one, and the version number used for each import is recorded in the final report, enabling the full auditability of all data transformations.

### Usability Considerations and Field Observations

No formal usability study was conducted in this work, and no standardized evaluation instruments (eg, system usability scale) were applied, as the primary focus was on the methodological and architectural design of the data integration pipeline. Nevertheless, the informal qualitative feedback collected during real-world deployment across several clinical centers provided contextual insights into the system use in operational environments. These observations suggested that the tool was perceived as easy to adopt in routine workflows and that it may reduce the complexity of data extraction–transformation–loading processes compared to manual approaches.

### Limitations and Considerations

Despite its advantages, FREDDEX is not without limitations. The system has been developed and evaluated using real-world clinical datasets, which constitute a meaningful stress test for any data integration pipeline. In practice, heterogeneous data entry conditions—particularly in free-text fields—may introduce special characters, encoding inconsistencies, or syntactic irregularities capable of disrupting automated processing. Although BaMaRa increasingly relies on structured fields and controlled vocabularies, residual variability inherent to routine clinical documentation remains an operational constraint.

To mitigate these risks, FREDDEX incorporates multiple safeguards, including error-handling mechanisms. As previously stated, when critical inconsistencies are detected, the import of the corresponding patient record is automatically blocked. Nevertheless, these technical safeguards do not eliminate the need for vigilant downstream data management, including routine quality control procedures, reconciliation checks, and periodic validation of transformation outputs against source data.

Another structural consideration lies in the need to maintain alignment with evolving upstream and downstream systems. FREDDEX operates at the intersection of BaMaRa and SKEZIA and must therefore accommodate changes originating from both environments, including modifications to the BaMaRa’s export structure, updates to SKEZIA’s API specifications, or the internal evolution of the FREDD e-CRF. Any structural modification affecting variables, formats, or validation constraints must be consistently reflected in the mapping configuration and transformation logic to preserve interoperability and semantic integrity. This requires the rapid identification and implementation of changes as soon as they are known to minimize disruption to ongoing data collection.

From a scalability perspective, FREDDEX has been stress-tested with datasets comprising up to 5000 patients in a single import batch. While such a volume already exceeds typical expected operational loads for RED registries—where routine imports are generally expected to involve 100 to 200 patients—it remains lower than the scale encountered in large university hospital environments managing very large patient cohorts.

Finally, FREDDEX is currently configured specifically for RED within a French national context, embedding business rules aligned with observed RED clinical practice and the variables defined within FREDD. While the externalized mapping configuration facilitates adaptation to alternative target data warehouses or disease domains, the validity and completeness of rule translation would require a strong reassessment if FREDDEX was deployed for other REDs, different RED networks, or in different national settings. The modular architecture lowers the technical barrier to such adaptation; however, domain-specific semantic alignment, workflow validation, and regulatory compliance checks would remain necessary.

### Future Directions

Several future directions emerge from this work, spanning interoperability with European infrastructures, methodological generalization, usability assessment, and support for longitudinal data integration.

- Integration with European infrastructures: REDgistry’s data model, developed around the Joint Research Center minimal European dataset, is based on the French SDM-MR but is not yet fully harmonized. FREDDEX’s modular mapping and transformation layers could be extended to act as a bridge between SDM-MR and the Joint Research Center model, enabling automated, traceable data conversion, and seamless interoperability across national and European registries, while reducing the need for complex manual transformations.
- Generalization to other domains: The modular structure of FREDDEX could be tested through a concrete use case in a different rare disease registry. Developing and deploying a FREDDEX instance for an alternative data source would allow for the evaluation of its modularity, adaptability of mapping rules, and robustness of the pipeline in a new context, providing practical insights for future scaling to other networks.
- Additional feedback from the field: Although informal field observations suggest that FREDDEX is rapidly appropriated by clinical data technicians with limited training and facilitates the data collection, a structured usability evaluation remains necessary. Conducting a formal assessment—potentially using standardized instruments such as the system usability scale—would provide the quantitative evidence of usability, identify unseen issues, and guide future improvements to enhance adoption and user experience.
- Longitudinal data tracking: As FREDD may eventually support a longitudinal patient follow-up, FREDDEX will need to adapt to enable incremental updates and synchronization of new or updated clinical data. This would require implementing mechanisms to detect previously imported patients, reconcile changes with existing records, and ensure that follow-up information is correctly linked to prior diagnostic blocks while preserving auditability and semantic consistency.

## Abbreviations

API: application programming interface
BaMaRa: Base Maladies Rares
BNDMR: Banque nationale de données maladies rares (National rare disease database)
CARGO: Centre des Affections Rares Génétiques et Ophtalmologiques
CNIL: Commission nationale de l’informatique et des libertés (the French Data Protection Authority)
e-CRF: electronic case report form
ERN: European reference network
FHIR: Fast Health care Interoperability Resources
FREDD: French rare eye disease database
GDPR: General Data Protection Regulations
HDW: health data warehouse
HPO: human phenotype ontology
PNMR: National Plan for Rare Diseases
RED: rare eye disease
SDM-MR: Minimum data set for rare diseases (Set de Données Minimum Maladies Rares)
